# Insulin and Human Serum Albumin Interactions with Core–Shell Fe_3_O_4_@SiO_2_ Nanoparticles Functionalized with Carboranes

**DOI:** 10.1021/acs.jpcb.5c00731

**Published:** 2025-06-28

**Authors:** Katarzyna Ludzik, Monika Marcinkowska, Barbara Klajnert-Maculewicz, Liangliang Huang, Monika Jazdzewska, Ilya V. Korolkov, Artem L. Kozlovskiy, Maxim V. Zdorovets, Natalia Jasiak

**Affiliations:** † Department of Physical Chemistry, 49602 University of Lodz, Lodz 90-236, Poland; ‡ Department of General Biophysics, Faculty of Biology and Environmental Protection, University of Lodz, Lodz 90-237, Poland; § School of Sustainable Chemical, Biological and Materials Engineering, 6187 University of Oklahoma, Norman, Oklahoma 73019, United States; ∥ Department of Experimental Physics of Condensed Phase, Faculty of Physics and Astronomy, 229195 Adam Mickiewicz University, Poznan 61-614, Poland; ⊥ The Institute of Nuclear Physics, Almaty 050032, Kazakhstan; # 565870 Eurasian National University, Astana 010008, Kazakhstan

## Abstract

In a biological medium, nanoparticles (NPs) can spontaneously interact with proteins, adsorb onto their surface, and cause conformational and orientation changes of the proteins. As a result, the protein function is influenced in a complex manner. Therefore, a detailed understanding of the nature and specificity of protein–nanoparticle interactions is crucial for the application of functional NPs in medicine. In the presented work, we studied the interactions of GMA-treated SiO_2_ NPs with the Fe_3_O_4_ core and attached carborane compounds (Fe_3_O_4_/TEOS/TMSPM/GMA/Carborane), designed for boron neutron capture therapy, with human serum albumin (HSA) and insulin. We combined different techniques: spectrofluorometry, circular dichroism spectroscopy, and isothermal titration calorimetry to address this issue. The results show that the adsorption of protein onto the NP surface is enthalpy–entropy-driven, with ensuing structural changes of the protein. As for albumin, the percentage of the α-helix structure in the protein is significantly reduced from 87.59 (free protein) to 40.9% for an NP concentration of 1.8 mg/mL, while the content of the β-sheet and random coil increases from 0.48 to 8.78% and from 11.93 to 50.32%, respectively. The interaction between NPs and small protein–insulin is weaker than that for HSA, confirming less negative Δ*H* and a 15% decrease in the α-structure content for the highest concentration of NPs. For both proteins, the exposure on Fe_3_O_4_/TEOS/TMSPM/GMA/Carborane affects the polarity of the microenvironment around Trp, which is consequently exposed to a more hydrophobic environment. Calculated values of the radius of gyration and the minimum distance between the proteins and the NPs indicate a stronger interaction and closer binding proximity to the NPs, corroborating experimental observations of the higher binding affinity of HSA to NPs.

## Graphical abstract

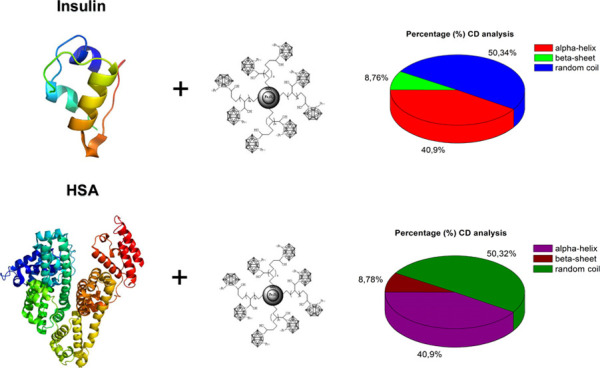

## 1 Introduction

Nowadays, materials science focuses on the production of new nanomaterials with desirable properties that can be used for various purposes in ecology, power generation, engineering, and biomedicine.^1–8^ Nanoparticles (NPs) with a surface modified by boron-rich compounds are attracting considerable attention due to their unique properties and potential application in boron neutron capture therapy (BNCT).^9,10^ BNCT is based on nuclear capture and fission following irradiation of nonradioactive boron-10 with neutrons, resulting in the production of α-rays and consequent cell destruction. Selective delivery of NPs to the tumor while avoiding normal cells seems to be the key aspect of BNCT application. Various carriers can be used, including magnetic NPs to enhance selective accumulation in tumor tissues.^11,12^ The main requirements for the use of nanostructures as containers for targeted drug delivery are the ability to transport drugs directly to the affected tissues or organs, reduction of side effects, nontoxicity of the carrier, and stability. The behavior of NPs in in vitro cellular systems sheds light on the mechanisms of interaction between NPs and biological systems and, consequently, allows us to predict the adverse effects of nanotechnology on living organisms. Due to their nano-size (similar to that of typical cellular components) and large surface-to-mass ratio, NPs can interact with biomolecules such as proteins, nucleic acids, etc. In biological media, proteins can adsorb to the NP surface, form complexes with the NP, and may undergo conformational changes. NPs are able to affect the secondary structures of proteins, causing changes in their function in cells.^13,14^ An uncontrolled formation of protein corona may alter the biodistribution of drugs, cause aggregation of NPs, and, in consequence, trigger unwanted immune responses.^14,15^ The properties of biomolecules (particularly their amphiphilic character) and the physicochemical parameters of NPs (e.g., size, surface polarity, surface defects, charge, and functionalization) determine their intermolecular interactions (e.g., electrostatic, dipol–dipol, hydrophobic, or hydrogen bonding). These interactions are responsible for protein corona formation.^15^ It has been observed that the adsorption of larger or less conformationally flexible proteins is suppressed by small round NPs as a consequence of their curvature.^16^ In addition, the interaction of protein with negatively charged NPs [where the charge is approximately −(30–50) mV] occurs immediately after contact, thereby reducing the charge to approximately −10 mV.^16^ Gold NPs have been described by Wangoo as materials that induce conformational changes in the structure of bovine serum albumin.^17^ An analysis of the protein binding affinity of titanium dioxide NPs revealed that positively charged R-groups and nonpolar aliphatic R-groups of amino acids play a dominant role in the formation of stable hydrogen bonds. The weak affinity of TiO_2_ NPs for amino acids with aromatic R-groups can be attributed to the fact that TiO_2_ NPs do not form a stable hydrogen bond due to resonance energy stabilization of the amino acid.^18^ According to Gheshlaghi, titanium dioxide causes conformational change and reduces polymerization of tubulin.^19^ Zahara et al. observed that the Fe NPs do not change the secondary structure of the hen egg white lysozyme but form hydrogen bonds and cause conformational changes in the tertiary structure on contact with the hen egg white lysozyme.^20^ The process of protein adsorption to NPs can be reduced by the introduction of a polymer layer shell, for example, polyethylene glycol, which prevents proteins from interacting directly with the NPs.^21^ NPs composed of hydrophobic materials, such as carbon and latex, exhibit a tendency to form a protein corona, primarily as a result of imperfect surface coverage.^22^ The amphiphilic character of the protein was found to facilitate adhesion to the NP surface, thereby reducing surface tension in cases where the coating was inadequate. It is worth mentioning that the concentration of NPs and protein properties can have also a meaningful impact on the protein corona and the binding affinity.^16^ Understanding the mechanisms of interaction between NPs and a biological system and the influence of shell modification on the behavior and properties of NPs is crucial for any future innovation in this field. In order to describe the mode of action between NPs and biomacromolecules, the interactions between NP proteins and nucleic acids should be considered, including those following structural modifications. The aim of this study was to investigate the interaction between GMA-treated SiO_2_ NPs and the Fe_3_O_4_ core with attached carborane compounds (Fe_3_O_4_/TEOS/TMSPM/GMA/Carborane), which are designed for use in BNCT alongside albumin and insulin. The investigation employed spectrofluorometry, circular dichroism (CD) spectroscopy, and isothermal titration calorimetry (ITC). The biocompatibility of the investigated NPs and their hemolytic properties were tested on human erythrocytes.

## 2 Materials and Methods

### 2.1 Nanoparticles

The procedure of Fe_3_O_4_/TEOS/TMSPM/GMA/Carborane synthesis and modification has been described before.^23^ The structure of NPs is presented in Figure 1.

**Figure 1:**
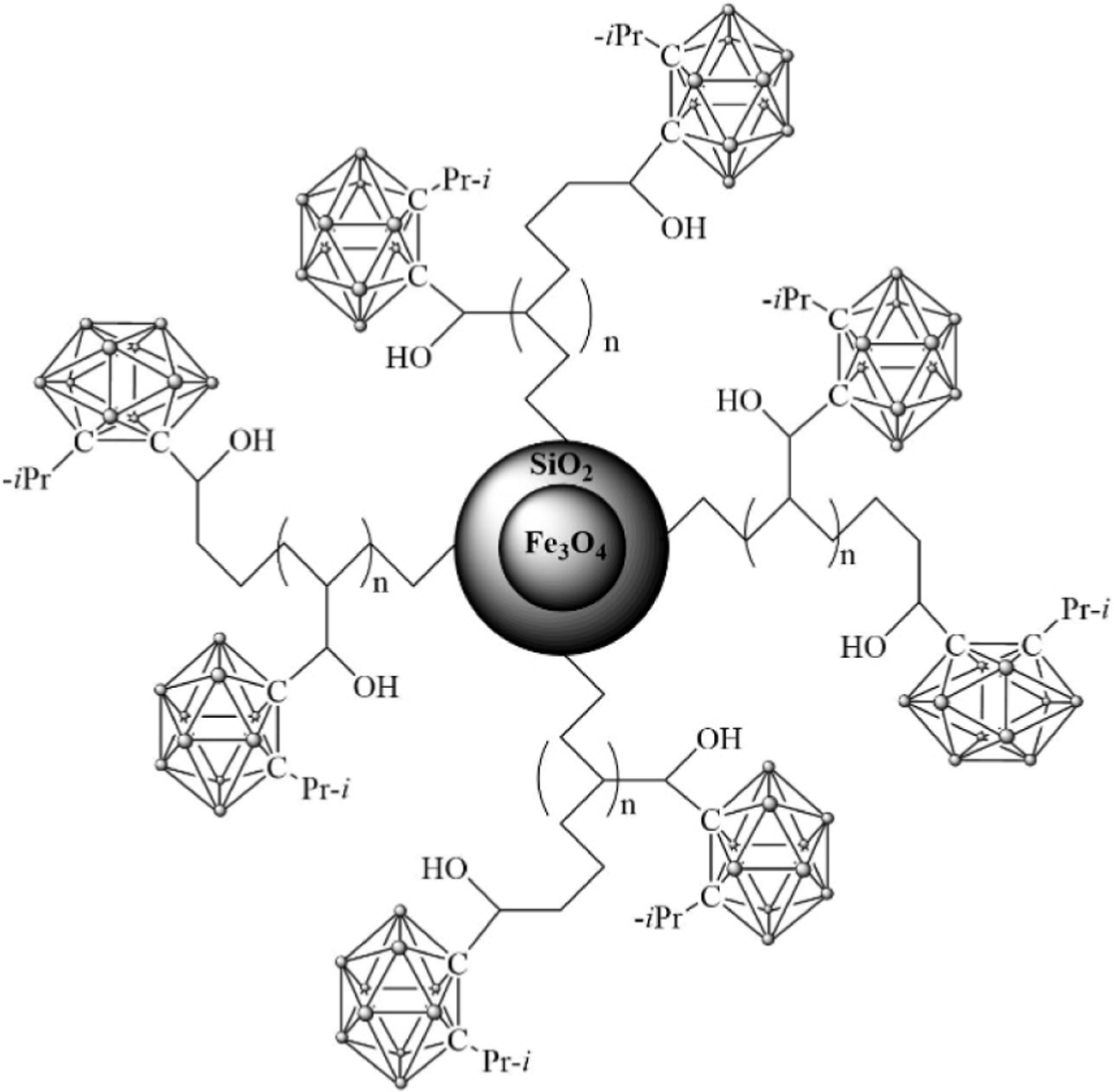
Structure of an Fe_3_O_4_/TEOS/TMSPM/GMA/Carborane NP.

### 2.2 Characterization

#### 2.2.1 FTIR—Fourier Transform Infrared Spectroscopy

The analysis was performed on a Fisher Scientific Nicolet I S5 FT-IR spectrometer (Waltham, MA, USA) at room temperature. Potassium bromide was used as a carrier for samples of NPs.

#### 2.2.2 Zeta Potential

The ξ-Potential of the NPs in a phosphate buffer (PB, pH 7.4) at 30 °C was determined by dynamic light scattering analysis using the Litesizer DLS 500 Anton Paar.

#### 2.2.3 Spectrofluorimetric Measurements of the Interactions between the Fe_3_O_4_/TEOS/TMSPM/GMA/Carborane and Proteins—Albumin and Insulin

The excitation and emission spectra were obtained on an LS 55 fluorescence spectrometer (PerkinElmer, Waltham, MA, USA) at a constant temperature of 25 °C. All samples were prepared in PBS buffer (10 mM, pH 7.4) and measured in quartz cuvettes. The excitation wavelength for human serum albumin (HSA) was set to 278 nm, and fluorescence spectra were recorded at between 295 and 450 nm. The excitation spectrum of this protein occurred in the range of 245–300 for the emission wavelength at 344 nm. The excitation wavelength for insulin was set to 273 nm, and fluorescence spectra were recorded between 295 and 350 nm. The excitation spectrum of this protein occurred in the range of 245–280 for the emission wavelength at 305 nm. Excitation and emission slits were 7.5 nm. The proteins solutions in a constant concentration of 0.5 mg/mL was titrated with NPs solution in final concentrations ranging from 0.1 to 1.5 mg/mL. The experiments were performed in three independent replicates.

#### 2.2.4 CD Studies on the Interactions between Fe_3_O_4_/TEOS/TMSPM/GMA/Carborane and Proteins—Albumin and Insulin

A JASCO J-815 CD spectropolarimeter (Jasco, Japan) was used for the CD spectra measurements. A stock solution of HSA and insulin (0.025 mg/mL) in 10 mM PB (pH 7.4) was prepared. The protein solutions were titrated with an NP solution in final concentrations ranging from 0.01 to 1.8 mg/mL. In all CD measurements, the samples were kept in a quartz cuvette. CD spectra were recorded with a path length of 0.05 cm in the wavelength range from 190 to 240 nm, at a temperature of 25 °C. Each spectrum was recorded as an average of three scans and collected with a scan speed of 50 nm min^–1^ and 0.5 nm bandwidth. Deconvolution of CD spectra was performed with the use of a neural network algorithm implemented in K2D3 computer software. The α-helix content in free proteins and in proteins treated with Fe_3_O_4_/TEOS/TMSPM/GMA/Carborane was calculated from mean residue ellipticity values using the K2D2 program.^24^

#### 2.2.5 Isothermal Calorimetric Titration Studies on the Interactions between Fe_3_O_4_/TEOS/TMSPM/GMA/Carborane and Proteins—Albumin and Insulin

ITC measurements were performed at 37 °C using a Microcal VP-ITC microcalorimeter. 10 mM PB (pH 7.4) with NPs at a concentration of ∼1.5 mg mL^–1^ was placed in a measuring cell (volume of 1.42 mL). The protein solutions (albumin and insulin with concentrations of 2.88 mg mL^–1^ and 1.99 mg mL^–1^, respectively) were titrated into the cell using an automatic syringe, which also acted as a stirrer to ensure homogeneous mixing of the titrated solution. The reference cuvette was filled with 10 mM PB. All measurements were replicated three times, and data processing was performed using Microcall Origin software. “Blank titrations” were performed by injecting the protein solution into the sample cell containing plain buffer. The enthalpograms obtained by protein titration of the NP solution were subtracted from the “blank titration” thermograms and fitted by a set of site models (Origin 7.0 with ITC data analysis, version 1.04.0012 OriginLab Corporation, Northampton, MA, USA).

#### 2.2.6 Hemolytic Activity

Blood from healthy donors was obtained from the Central Blood Bank in Lodz. Erythrocytes (red blood cells) were separated from blood plasma and leukocytes by centrifugation (2500*g*, 10 min, 4 °C) and washed three times with PBS (phosphate buffered saline; pH 7.4). To study the effect of hemotoxicity, NPs at concentrations in the range of 10–200 mg mL^–1^ were added to an erythrocyte suspension with 2% hematocrit and incubated at 37 °C for 3 and 24 h. The suspensions were then centrifuged (2500*g*, 10 min, 4 °C). Hemolysis was determined by measuring the hemoglobin content of the supernatant at 540 nm. The results were expressed as a percentage of the control probe (hemolysis after addition of water).

#### 2.2.7 Computational Analysis of the Protein–NP Interaction

A custom Python script was developed in-house to computationally analyze the interactions of HSA and insulin with core–shell Fe_3_O_4_@SiO_2_ NPs. Protein atomic coordinates were imported from crystal structures available in the Protein Data Bank (HSA: PDB ID 1ao6; insulin: PDB ID 3i40) using the Biopython library. NPs were modeled as simplified representations with randomly distributed atoms confined within a cubic volume (box size: 20 nm) to approximate their surfaces. To simulate minimal conformational fluctuations upon interaction with NPs, molecular-dynamics-inspired random positional perturbations (Gaussian distribution, σ = 0.1 nm) were introduced. Structural parameters, including RMSD, *R*_g_, and minimum interaction distances, were calculated to quantitatively evaluate the interaction strength and structural integrity. The Python script is included in the Supporting Information.

## 3 Results and Discussion

### 3.1 FTIR Spectra

Figure 2 represents the obtained FTIR spectra for Fe_3_O_4_/TEOS/TMSPM/GMA/Carborane and TEOS/TMSPM/GMA/Carborane.

**Figure 2:**
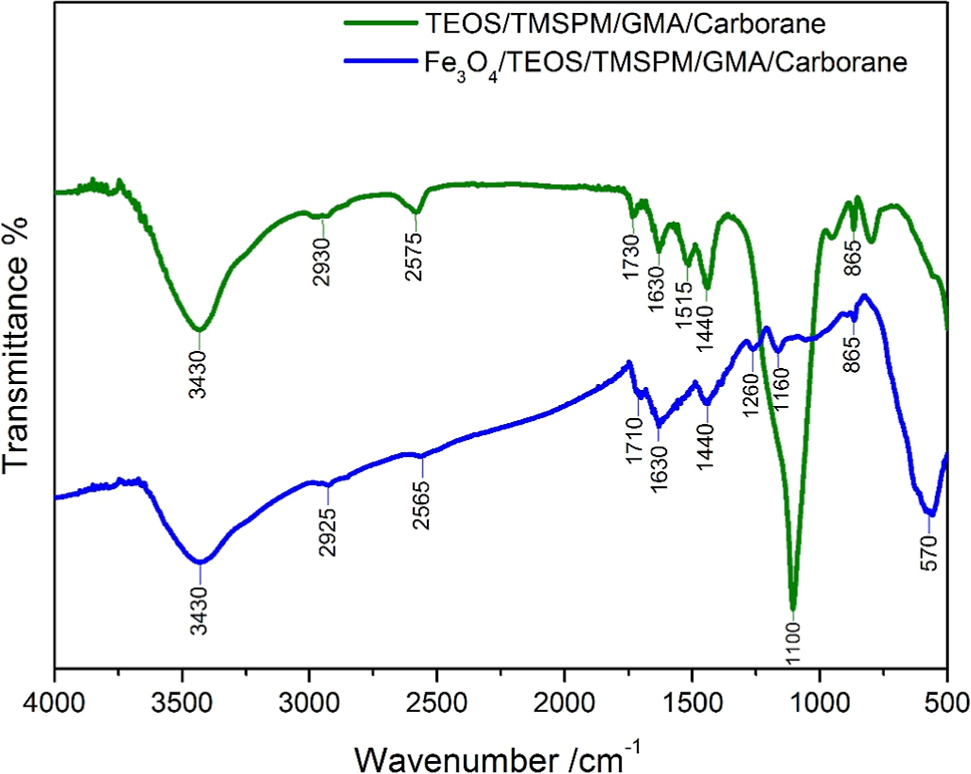
FTIR spectra for Fe_3_O_4_/TEOS/TMSPM/GMA/Carborane and TEOS/TMSPM/GMA/Carborane.

The spectra shown for modified NPs with and without a magnetic core are similar. The main difference appears at 800 cm^–1^, 1100 cm^–1^, and 570 cm^–1^. In the case of Fe_3_O_4_/TEOS/TMSPM/GMA/Carborane, a peak at 570 cm^–1^ related to Fe–O–Fe results in the broadening of peaks at 1100 cm^–1^ and 800 cm^–1^ (reflecting Si–O–Si symmetric and asymmetric stretching vibration bonding). The broad peak at 3430 cm^–1^ and the peak at 1630 cm^–1^ are attributed to υ­(O–H) and δ­(O–H) stretching, respectively. CH_2_ stretching vibrations are observed at 2920 cm^–1^. Typical for carboranes, asymmetric and symmetric C–H stretching vibrations appear in the 3010 cm^–1^ region.^25^ Peaks at 2565 and 2575 cm^–1^ correspond to υ­(B–H) vibrations. Peaks at 1730 and 1710 cm^–1^ correspond to C=O stretching vibrations. CH_3_ asymmetric stretching and CH_2_ stretching peaks are visible at 1440 and 1515 cm^–1^. C–O–C ether stretching bonds and C–O–C weak ring stretching bending of the epoxide ring are visible at 1160 cm^–1^ and 1260 cm^–1^, respectively. 865 cm^–1^ is associated with Si–CH_3_ vibrations (rocking vibrations).

### 3.2 Spectrofluorometric Spectra of the Excitation and Emission Spectra

Protein fluorescence is associated with the presence of aromatic amino acids in its chain: tryptophan (Trp), tyrosine (Tyr), and phenylalanine (Phe) residues. However, to be more precise, the fluorescence of proteins is mainly contributed by Trp because the Phe residues have a very low quantum yield, and the fluorescence of Tyr is almost completely quenched. The fluorescence spectrum of proteins is sensitive to the microenvironment, which is associated with a change in the polarity around Trp residues. A shift in the fluorescence maximum to the right or left indicates a change in the chromophore environment. A blue shift of the emission maximum indicates that the amino acid residues are in a more hydrophobic environment and, therefore, are less exposed to the solvent. A red shift of the emission maximum indicates that the amino acid residues are in a more polar environment and are more exposed to the solvent.^26^

The fluorescence spectra of HAS (emission wavelength 344 nm) and insulin (emission wavelength 305 nm) in the absence or with an increasing concentration of Fe_3_O_4_/TEOS/TMSPM/GMA/Carborane nanocomposites are shown in Figure 3a,b, respectively.

**Figure 3:**
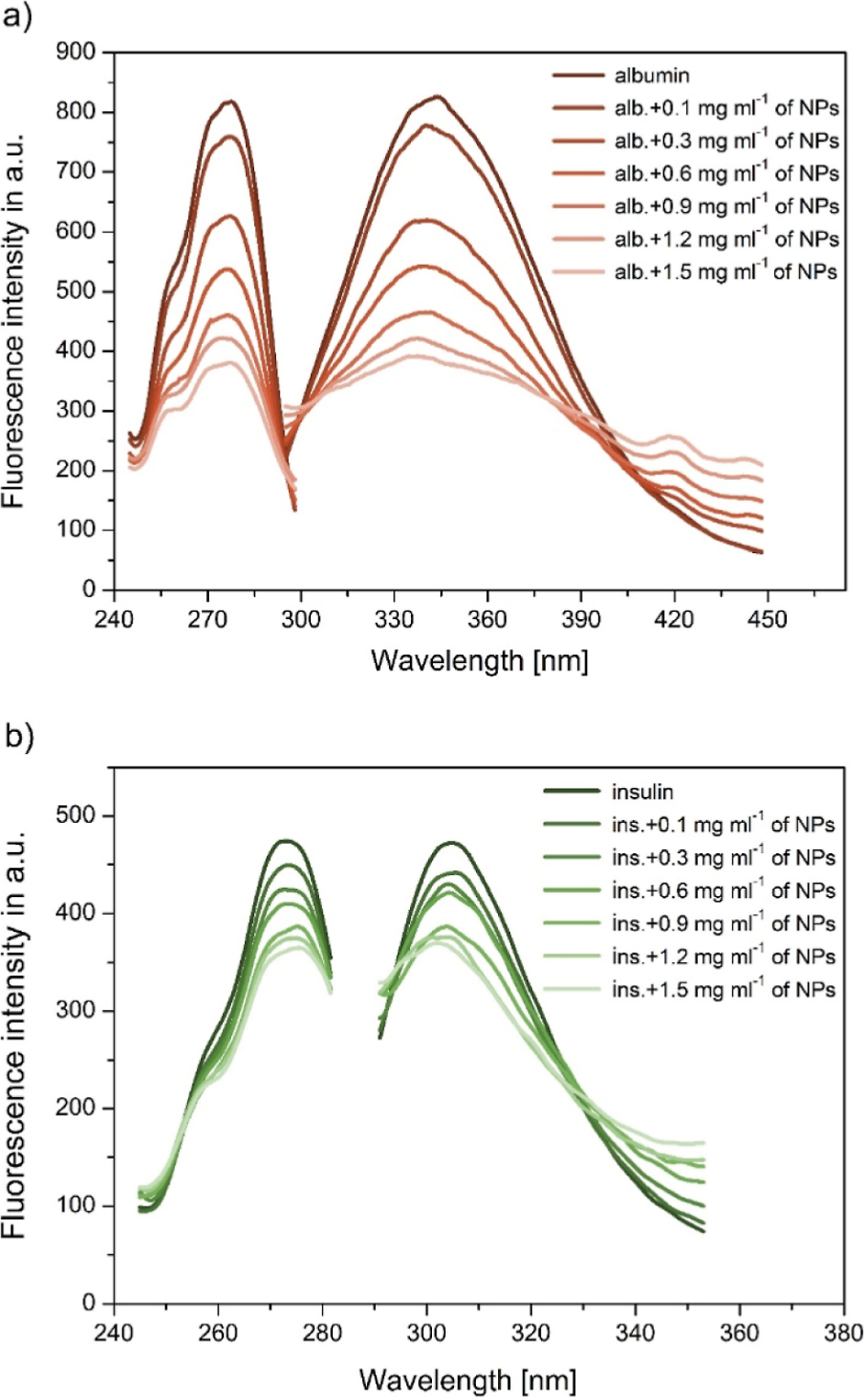
Excitation and emission spectra of human albumin 3a and insulin 3b in the absence or presence of Fe_3_O_4_-polymer nanocomposites. The concentration of protein was 0.5 mg mL^–1^ while the Fe_3_O_4_-polymer nanocomposite concentration corresponded to 0, 0.1, 0.3, 0.6, 0.9, 1.2, 1.5 mg mL^–1^. PB, pH = 7.4, 298.15 K, λ_ex_ = 278 nm and λ_ex_ 273 nm, respectively.

The fluorescence intensity of the Trp residue is reduced and demonstrates a slight blue shift. The results suggest that the polarity microenvironment around the Trp residue decreases, and the Trp residue is exposed to a more hydrophobic environment. The addition of Fe_3_O_4_/TEOS/TMSPM/GMA/Carborane leads to a significant decrease in fluorescence intensity. These results indicate that Fe_3_O_4_/TEOS/TMSPM/GMA/Carborane nanocomposites interact with HSA and insulin and quench their intrinsic fluorescence.^27^

### 3.3 CD Studies of the Interactions between Fe_3_O_4_/TEOS/TMSPM/GMA/Carborane and Proteins—Albumin and Insulin

CD spectroscopy is a structural biology technique used to study the structure of proteins, polypeptides, and peptides. In the far ultraviolet, the spectra of these molecules are dominated by n–π* and π–π* transitions of amide groups, which then influence the geometries of polypeptide backbones and the secondary structures of the spectra. To confirm the influence of Fe_3_O_4_/TEOS/TMSPM/GMA/Carborane on the secondary structure of proteins, CD spectra were recorded in the presence of the compound at various concentrations. The obtained spectra are shown in Figure 4a,b.

**Figure 4:**
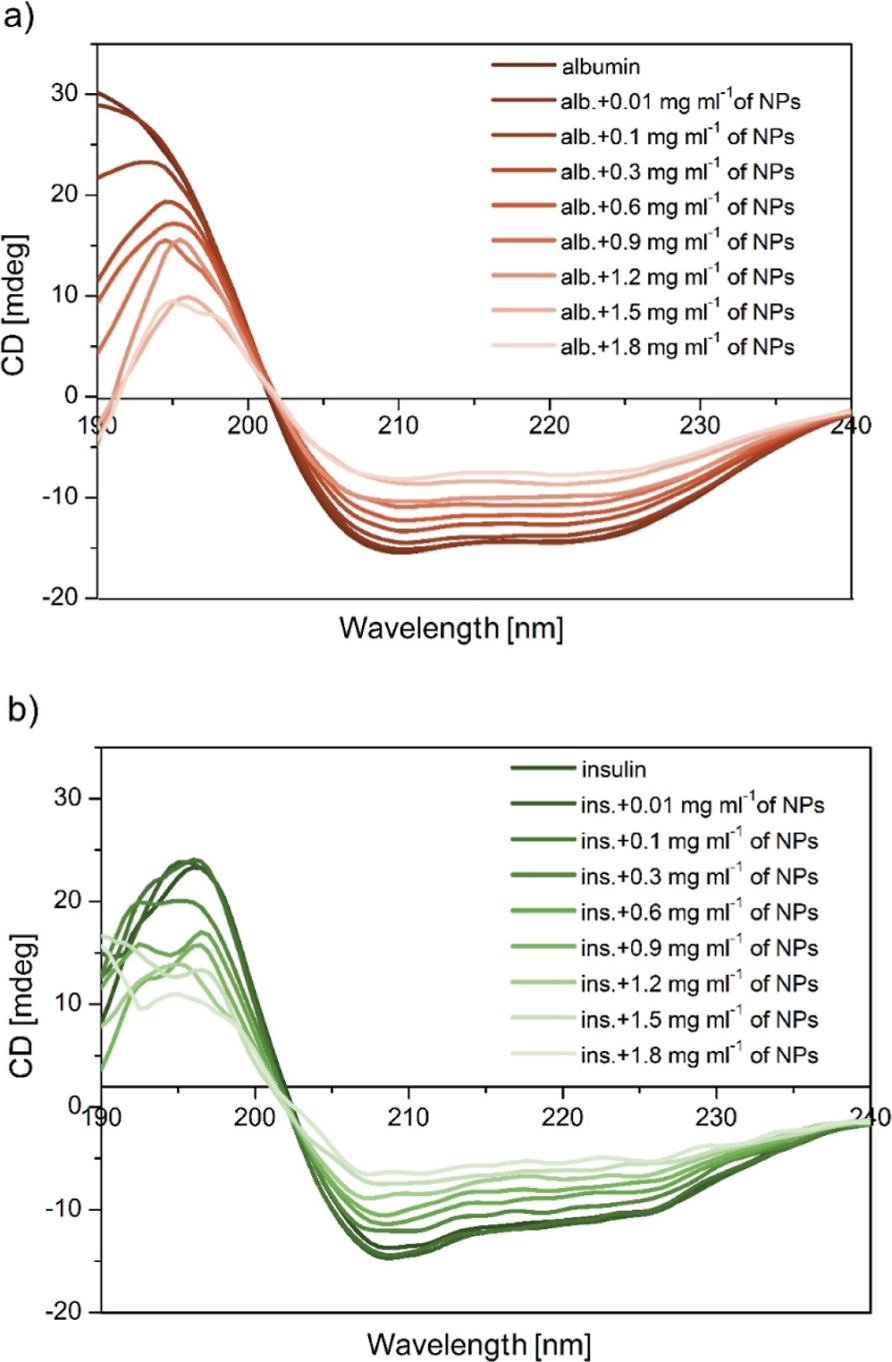
CD spectra of the protein—Fe_3_O_4_/TEOS/TMSPM/GMA/Carborane nanocomposite (4a HAS, 4b insulin). The protein concentration equaled 0.025 mg mL^–1^ and the Fe_3_O_4_–polymer nanocomposite concentration ranged from 0.01 to 1.8 mg mL^–1^, respectively.

The CD spectra of native proteins have two negative bands typical of the α-helix structure.^28^ The negative peaks between 209 and 210 nm and around 223–225 nm are associated with n–π* transfer for the peptide bond of the α-helix.^29^ Negative band intensities were observed to fatten after the addition of Fe_3_O_4_/TEOS/TMSPM/GMA/Carborane nanocomposites, indicating significant changes in the protein secondary structure, specifically that of albumin. These results suggest that the investigated nanocomposites interact with the proteins by inducing conformational changes, with a significant loss of the α helix structure in the tested system.

The spectrum of insulin, characterized by two negative minima at 209 and 222 nm, is characteristic for the α-structure.^30^ CD spectra of insulin in the presence of Fe_3_O_4_/TEOS/TMSPM/GMA/Carborane nanocomposites were obtained at increasing concentrations of the component. The addition of nanocomposites to insulin caused slight changes in the secondary structure of the hormone compared to that of albumin. The similarity between the CD spectrum shapes of free insulin and the nanocomposite:insulin complex suggests that the structure of the complex remained largely unchanged.^26,31^ With an increasing concentration of NPs, the percentage of the α-helix structure in the protein was observed to be reduced significantly from 87.59 (free protein) to 40.9% (1.8 mg mL^–1^ of NPs) for human albumin, while the content of the β-sheet and random coil increased from 0.48 to 8.78% and from 11.93 to 50.32%, respectively. The results for insulin also showed a decrease in the α-structure content, although not as significant, from 67.22% (free protein) to 52.46% (at the highest concentration of NPs). Consequently, the content of the β-sheet structure and random coil for insulin increased from 2.22 to 9.17% and from 30.56 to 38.37%, respectively. The decrease in α-helical structures indicates that the binding of Fe_3_O_4_/TEOS/TMSPM/GMA/Carborane nanocomposites with HSA and insulin causes their conformational changes and the loss of α-helical stability.^27^ An increase in the Fe_3_O_4_/TEOS/TMSPM/GMA/Carborane concentration was observed to be accompanied by a significant reduction in the percentage of the α-helix structure in the protein from 87.59 (free protein) to 40.9% (1.8 mg mL^–1^ of Fe_3_O_4_/TEOS/TMSPM/GMA/Carborane) for human albumin, while the content of the β-sheet and random coil increased from 0.48 to 8.78% and from 11.93 to 50.32%, respectively, Figure 5. The protein secondary structure change or loss upon adsorption on NPs is frequently observed for protein–NPs systems and can introduce heterogeneity in the adsorption process.^32^ In the literature, systems exhibiting strong protein conformational changes on NPs are often reported as the preferred system to form multilayers or NP agglomerates induced by protein adsorption.^21^

**Figure 5:**
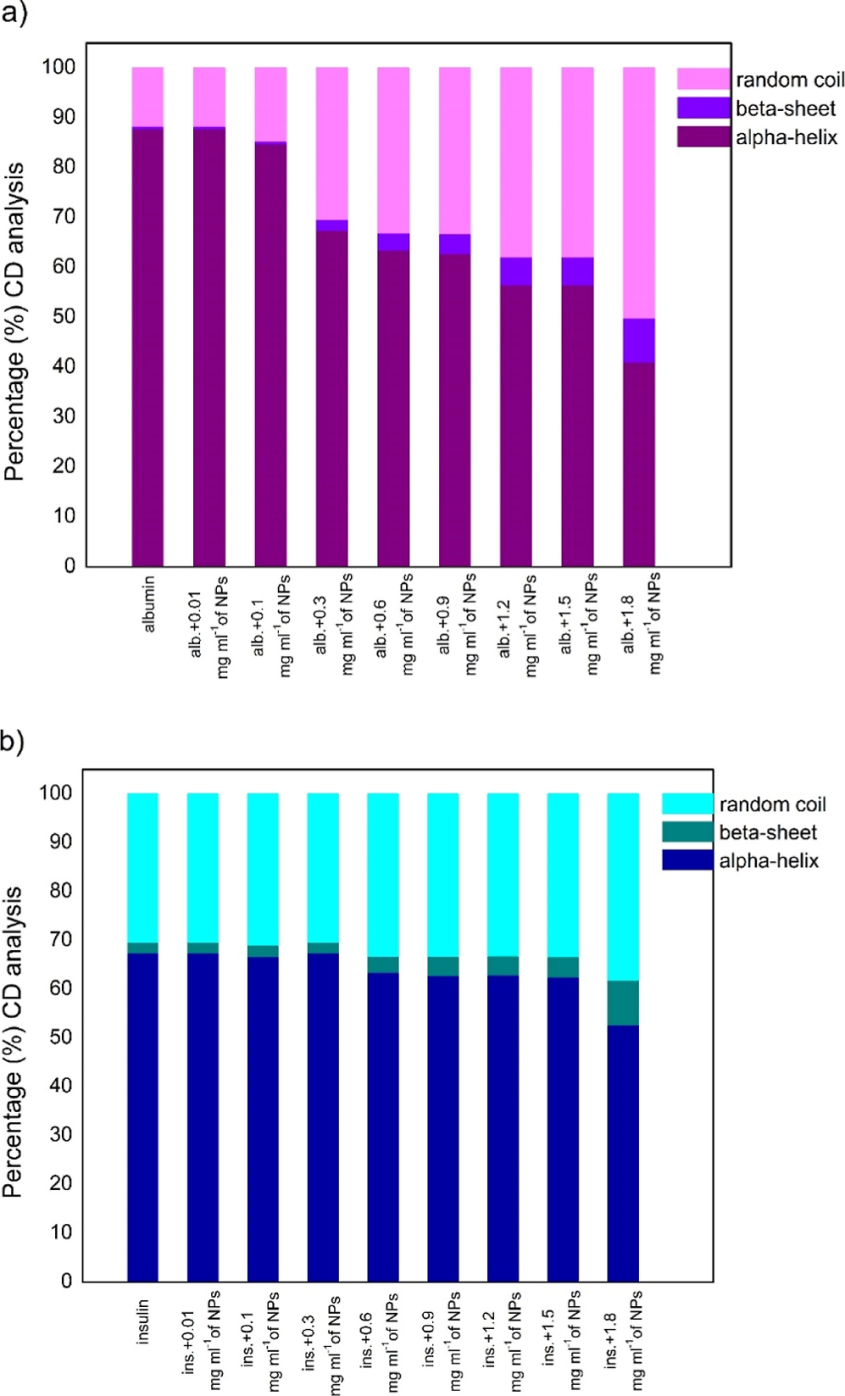
Percentage of the α-helix, β-sheet, and random coil content in free protein (5a HAS, 5b insulin) and Fe_3_O_4_/TEOS/TMSPM/GMA/Carborane complexes with an increasing concentration of the nanocomposite.

The results for insulin also showed a decrease in the α-structure content, although not as significant, from 67.22% (free protein) to 52.46% (for the highest concentration of NPs), Figure 5b. Consequently, the content of the β-sheet structure and random coil for insulin increased from 2.22 to 9.17% and from 30.56 to 38.37%, respectively. The decrease in α-helical structures indicates that the binding of Fe_3_O_4_/TEOS/TMSPM/GMA/Carborane with HSA and insulin causes their conformational changes and the loss of α-helical stability.^27^

### 3.4 Isothermal Titration Calorimetric Studies of the Interactions between Fe_3_O_4_/TEOS/TMSPM/GMA/Carborane and Proteins—Albumin and Insulin

Microcalorimetric analyses were performed to obtain quantitative information about the binding of NPs with albumin and insulin. The enthalpograms obtained are shown in Figure 6.

**Figure 6:**
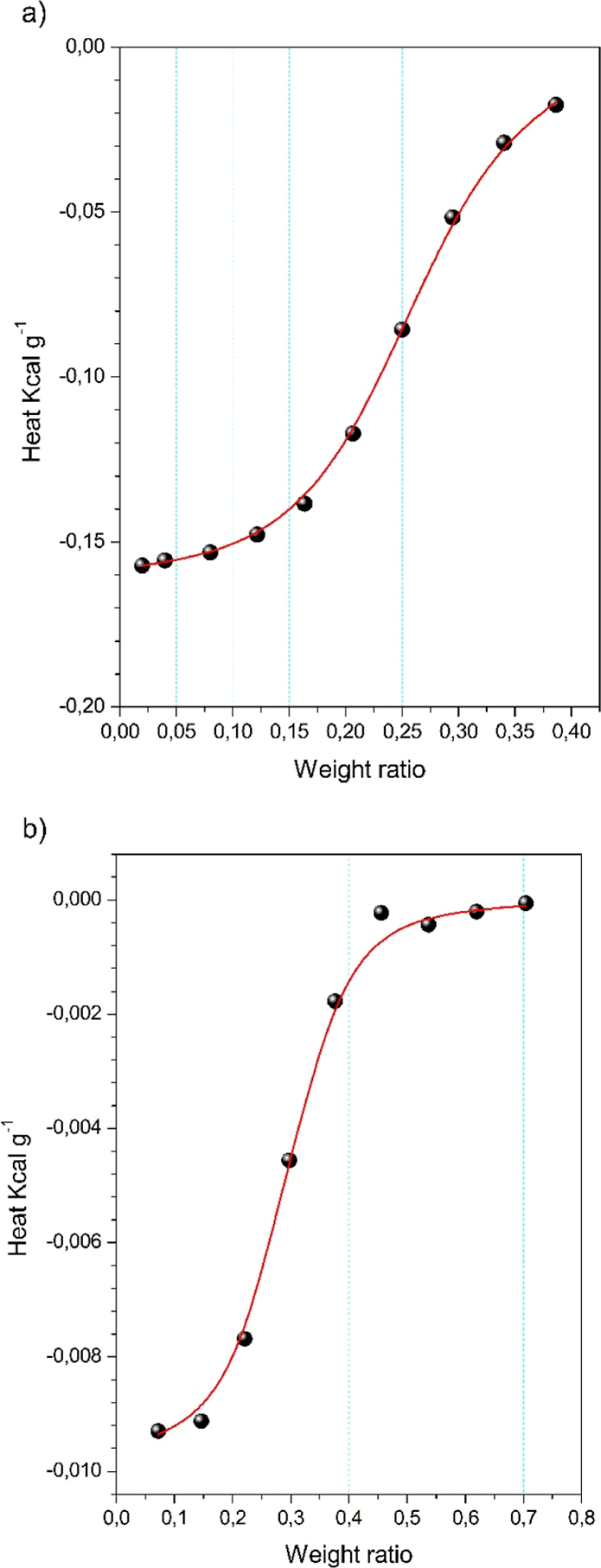
Integrated normalized heats from each addition of a protein (6a HSA, 6b insulin) into NPs solution corrected by the heats of dilution together with a fit corresponding to an independent binding model—single-site (straight line).

The calorimetric data were found to fit a single-site model. The interaction parameters shown in Table 1 indicate that in both cases, the interactions were driven by overall exothermic (−Δ*H*) and entropic (+Δ*S*) processes. The interaction between proteins and NPs is occurring against the Coulomb forces as their ξ-potentials are negative at pH = 7.4 (measured ξ for NP-s at 30 °C = −12.4 mV, HSA = 17 mV, and insulin = −5.81 mV).^33,34^ Due to the lack of Coulomb attractive forces, which are endothermic, negative values of Δ*H* correspond to hydrophobic interactions between hydrophobic protein domains with i–Pr containing parts of NPs.^33^ The positive values of Δ*S* can be interpreted mainly as shape changes of the protein. CD results indicate that the binding between NPs and the studied proteins induces conformational changes of the protein—loosening of the α-helix structure and, as a result, reduced stability of the protein. These changes are manifested as positive changes in entropy. Stronger interactions between NPs and albumin in comparison with insulin and a more notable decrease of the α-structure content confirm a more negative value of Δ*H* and a higher value of Δ*S*, Table 1.

**Table 1:** Thermodynamic Parameters of Protein–NP Interactions Estimated from ITC Using a Single-Site Binding Model^a^

type of protein	Δ*H* cal g^–1^	Δ*S* cal g^–1^ K^–1^	*K*	*N*
albumin	–163.4 ± 0.47	21.8	7.55 × 10^4^ ± 2.00 × 10^3^	0.246 ± 0.00057
insulin	–9.706 ± 1.1	20.4	2.87 × 10^4^ ± 5.27 × 10^3^	0.262 ± 0.00384

a Δ*H*—enthalpy, Δ*S*—entropy, *K*—binding constant, *N*—number of sites.

The NPs studied showed a higher binding affinity for albumin (higher *K* values) compared to insulin. This tendency can be interpreted in terms of the size of the protein, which primarily influences the conformational change, folding, and unfolding, and then the coating of the surface. Larger proteins induce greater changes through their contact with the NP.^35,36^ In addition, the thermodynamic affinity of large proteins is higher than that of smaller proteins due to the greater susceptibility of the structure to conformational changes, which is reflected in a larger contact area and thus a greater number of binding points per protein molecule.^37^ In the case of albumin, which is classified as a medium protein, a conformational change is observed after contact with the surface, leading to more thermodynamically favorable structures, which is even more pronounced in the case of small proteins. In this work, we have attempted to calculate the root mean square deviation—RMSD, radius of gyration—*R*_g_, and minimum distance—MD between HSA and insulin to the NP using a Python code and compared the obtained results with experimental methods, Table 2. It is important to emphasize that the reported *R*_g_ values specifically refer to the protein structures of HSA and insulin. These values were calculated solely on the basis of the atomic coordinates of each protein obtained from crystal structures (PDB IDs: HSA—1ao6; insulin—3i40). Neither the NPs nor the ligand molecules were included in the computation of *R*_g_ values. This computational approach provides direct insight into the spatial distribution and structural compactness of the proteins. Consequently, the difference in *R*_g_ values between HSA (34.95 nm) and insulin (20.77 nm) reflects their inherent structural complexity and size, independent of the ligand or NP characteristics.

**Table 2:** Calculated Values of the Root Mean Square Deviation—RMSD, the Radius of Gyration—*R*_g_, and the Minimum Distance—MD between Albumin and Insulin to the NP

type of protein	RMSD nm	*R*_g_ nm	MD nm
albumin	0.1716	34.95	0.18
insulin	0.1718	20.77	4.46

These low RMSD values indicate that the binding of core–shell NPs to HSA and insulin does not significantly distort the overall structural integrity of the proteins during the interaction process. The larger *R*_g_ for HSA compared to insulin is consistent with the fact that HSA is a larger protein with greater structural complexity, resulting in a greater spatial distribution of its atoms. The smaller minimum distance for HSA indicates a stronger interaction and closer binding proximity to the NP, confirming experimental observations of a higher binding affinity of HSA for the NPs studied.

### 3.5 Biological Safety of the Investigated NPs

To assess the biocompatibility of the NPs studied, the hemolytic properties were tested on human erythrocytes. For a short incubation time, the percentage of hemolysis for each compound concentration was compared with a negative control (no NP). For 24 h incubation, the percentage of hemolysis increased slightly from 4.03% (negative control) to 5.59% for the highest compound concentration Figure 7.

**Figure 7:**
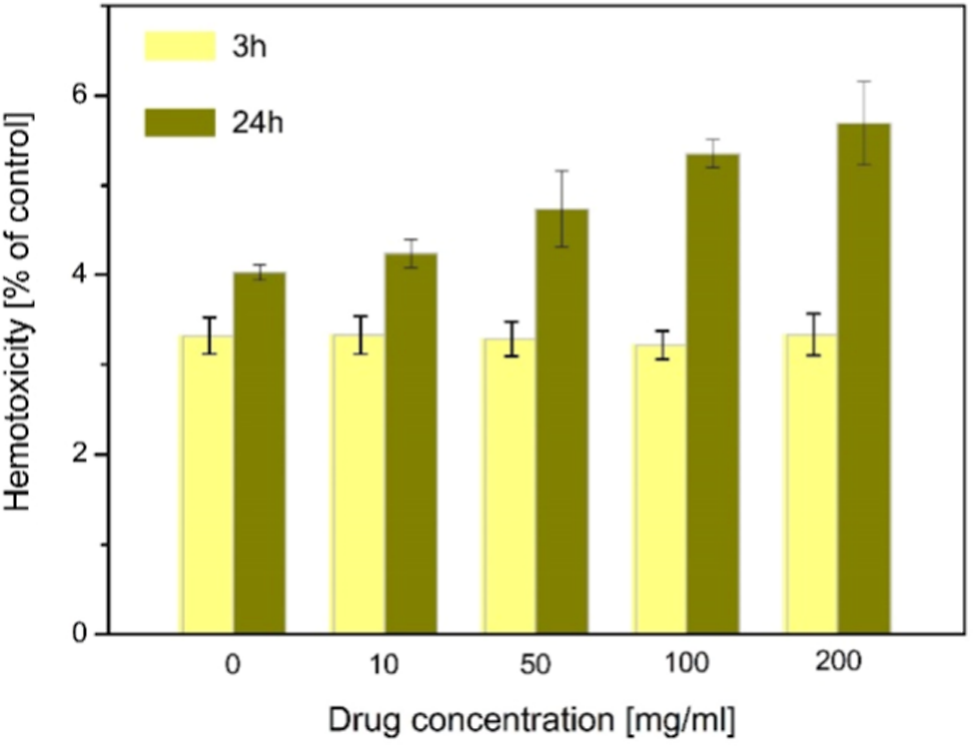
Effect of hemotoxicity and Fe_3_O_4_/TEOS/TMSPM/GMA/Carborane complexes at concentrations in the range of 10–200 mg mL^–1^ added to a red blood cell suspension of 2% hematocrit and incubated at 37 °C for 3 and 24 h. The positive control is 100% damage.

The mechanism of cytotoxicity of Fe_3_O_4_ nanomaterials is based on: induction of oxidative stress by excessive levels of reactive oxygen species, release of toxic Fe^2+^ ions, and disruption of transport (ionic or electronic) within a cell membrane.^16,38,39^ In addition, SiO_2_ in the shell can cause membrane rupture and protein unfolding.^16,40^ However, the NP tested did not damage the erythrocytes, even at a high concentration (200 mg mL^–1^). This suggests that the shell of modified NPs mitigates toxicity and that the NPs are therefore suitable for a wide safety margin in blood contact applications (Figure 7).

## 4 Conclusions

The investigated NPs with a surface modified by carboranes demonstrated a negative charge in PB; therefore, they interacted with proteins HSA and insulin (negative zeta potential) against Coulomb forces. The exothermic enthalpy change indicated hydrophobic interactions between hydrophobic protein domains with i–Pr containing parts of NPs. In consequence, the polarity microenvironment around the Trp residue decreased, and the Trp residue was exposed to a more hydrophobic environment. Positive values of Δ*S* and CD results indicate that the binding between NPs and the studied proteins induces conformational changes of the protein—loosening of the α-helix structure and, as a result, reduced stability of the protein.

The percentage of the α-helix structure in HSA is significantly reduced from 87.59% (free protein) to 40.9% for an NP concentration of 1.8 mg/mL, while the content of the β-sheet and random coil increases from 0.48% to 8.78% and from 11.93% to 50.32%, respectively. A decrease in the α-structure content for insulin is not as significant, from 67.22% (free protein) to 52.46% (at the highest concentration of NPs). CD results, thermodynamic parameters, and calculated parameters indicated a higher binding affinity for albumin compared to a small protein—insulin—due to the greater susceptibility of the structure to conformational changes, which is reflected in a larger contact area and thus a greater number of binding points per protein molecule. The NPs tested did not induce toxic effects on the erythrocytes even at a high concentration.

## Supplementary Material



## Notes

Python code for HSA–insulin–nanoparticle interaction calculations (PDF)
