# Supplementary material for: Insulin and Human Serum Albumin Interactions with Core–Shell Fe3O4@SiO2 Nanoparticles Functionalized with Carboranes
Source: J Phys Chem B. 2025 Jun 28;129(27):6757–64. doi: 10.1021/acs.jpcb.5c00731 (PMC12257507; doi:10.1021/acs.jpcb.5c00731)
Supplement: Supplementary file 1 [file jp5c00731_si_002.pdf]

```
# HSA-Insulin-Nanoparticle Interaction
import os
import numpy as np
import random
import matplotlib.pyplot as plt
from mpl_toolkits.mplot3d import Axes3D
from Bio.PDB import PDBParser
from scipy.spatial.distance import cdist
from scipy.ndimage import gaussian_filter1d
import matplotlib.animation as animation

# Function to check and load PDB file with specific path for local execution

def load_pdb_file(file_path, description):
    parser = PDBParser(QUIET=True)
    structure = parser.get_structure(description, file_path)
    print(f"Loaded {description} from {file_path}")
    return np.array([atom.coord for atom in structure.get_atoms()])

# Step 1: Load Protein Structures from PDB Files
try:
    positions_hsa = load_pdb_file("/Users/PDB/HSA-1ao6.pdb", "HSA")
    positions_insulin = load_pdb_file("/Users/PDB/Insulin-3i40.pdb", "Insulin")
except FileNotFoundError as e:
    print(e)
    print("Ensure the PDB files are placed in the specified path.")
    exit(1)

# Step 2: Generate Simplified Nanoparticle Model
num_atoms_nanoparticle = 500
box_size = 20.0
positions_nanoparticle = np.random.rand(num_atoms_nanoparticle, 3) * box_size

# Step 3: Combine Protein and Nanoparticle Systems
positions_hsa_combined = np.vstack((positions_hsa, positions_nanoparticle))
positions_insulin_combined = np.vstack((positions_insulin,
positions_nanoparticle))

# Step 4: Compute RMSD and Radius of Gyration
def compute_rmsd(reference_positions, trajectory_positions):
    """Compute RMSD between reference and trajectory positions."""
    return np.sqrt(np.mean(np.sum((trajectory_positions -
reference_positions)**2, \
    axis=1)))

def compute_radius_of_gyration(positions):
    """Compute the radius of gyration for a set of positions."""
    centroid = np.mean(positions, axis=0)
    return np.sqrt(np.mean(np.sum((positions - centroid)**2, axis=1)))
```

```
# Step 5: Compute Protein-Nanoparticle Interaction Distances
def compute_min_distances(protein_positions, nanoparticle_positions):
    """Compute the minimum distances between each protein atom and the
nanoparticle."""
    distances = cdist(protein_positions, nanoparticle_positions)
    min_distances = np.min(distances, axis=1)
    return min_distances

min_distances_hsa = compute_min_distances(positions_hsa, positions_nanoparticle)
min_distances_insulin = compute_min_distances(positions_insulin,
positions_nanoparticle)

# Step 6: Compute Radial Distribution Function (RDF)
def compute_rdf(protein_positions,nanoparticle_positions,bin_width=0.05,\
    max_distance=10.0):
    """Compute RDF between protein and nanoparticle atoms."""
    distances = cdist(protein_positions, nanoparticle_positions).flatten()
    bins = np.arange(0, max_distance + bin_width, bin_width)
    rdf, _ = np.histogram(distances, bins=bins, density=True)
    bin_centers = (bins[:-1] + bins[1:]) / 2
    return bin_centers, rdf

rdf_bins_hsa, rdf_values_hsa = compute_rdf(positions_hsa,
positions_nanoparticle)
rdf_bins_insulin, rdf_values_insulin = compute_rdf(positions_insulin, \
positions_nanoparticle)

# Apply Gaussian smoothing to RDF results
rdf_values_hsa_smoothed = gaussian_filter1d(rdf_values_hsa, sigma=2)
rdf_values_insulin_smoothed = gaussian_filter1d(rdf_values_insulin, sigma=2)

# Save RDF results
np.savetxt('rdf_hsa.txt', np.column_stack((rdf_bins_hsa,
rdf_values_hsa_smoothed)), \
header="Distance\tRDF")

np.savetxt('rdf_insulin.txt', np.column_stack((rdf_bins_insulin, \
rdf_values_insulin_smoothed)), header="Distance\tRDF")

# Plot RDF results (Raw and Smoothed)
plt.figure(figsize=(10, 6))
plt.plot(rdf_bins_hsa, rdf_values_hsa, label='HSA-Nanoparticle RDF (Raw)', \
         linestyle='--', color='blue')

plt.plot(rdf_bins_hsa, rdf_values_hsa_smoothed, \
        label='HSA-Nanoparticle RDF (Smoothed)', color='blue')

plt.plot(rdf_bins_insulin, rdf_values_insulin, \
        label='Insulin-Nanoparticle RDF (Raw)', linestyle='--', color='green')
```

```
plt.plot(rdf_bins_insulin, rdf_values_insulin_smoothed, \
        label='Insulin-Nanoparticle RDF (Smoothed)', color='green')

plt.xlabel('Distance (nm)')
plt.ylabel('RDF')
plt.title('Radial Distribution Function (RDF) - Raw and Smoothed')
plt.legend()
plt.grid(True)
plt.savefig('rdf_plot_smoothed.png')
plt.show()

# Step 7: Use initial positions as reference
reference_hsa = positions_hsa_combined.copy()
reference_insulin = positions_insulin_combined.copy()

# Simulate random movement (for demonstration purposes)
num_frames = 100  # Number of frames for the animation
trajectories_hsa = [positions_hsa_combined + \
np.random.normal(0, 0.1, positions_hsa_combined.shape) * \
(i / num_frames) for i in range(num_frames)]

trajectories_insulin = [positions_insulin_combined + \
np.random.normal(0, 0.1, positions_insulin_combined.shape) * \
(i / num_frames) for i in range(num_frames)]

# Compute RMSD and radius of gyration for the final frame
rmsd_hsa = compute_rmsd(reference_hsa, trajectories_hsa[-1])
rmsd_insulin = compute_rmsd(reference_insulin, trajectories_insulin[-1])

radius_gyration_hsa = compute_radius_of_gyration(trajectories_hsa[-1])
radius_gyration_insulin = compute_radius_of_gyration(trajectories_insulin[-1])

# Step 8: Save Results
np.savetxt('rmsd_hsa.txt', [rmsd_hsa])
np.savetxt('rmsd_insulin.txt', [rmsd_insulin])
np.savetxt('radius_gyration_hsa.txt', [radius_gyration_hsa])
np.savetxt('radius_gyration_insulin.txt', [radius_gyration_insulin])
np.savetxt('min_distances_hsa.txt', min_distances_hsa)
np.savetxt('min_distances_insulin.txt', min_distances_insulin)

# Display Results
print("Simulation completed. Results saved.")
print("RMSD (HSA):", rmsd_hsa)
print("RMSD (Insulin):", rmsd_insulin)
print("Radius of Gyration (HSA):", radius_gyration_hsa)
print("Radius of Gyration (Insulin):", radius_gyration_insulin)
print("Min Distance (HSA to Nanoparticle):", np.min(min_distances_hsa))
print("Min Distance (Insulin to Nanoparticle):", np.min(min_distances_insulin))

# Step 9: Create Trajectory Animations and Save as MP4
```

```
# HSA Animation
fig_hsa = plt.figure(figsize=(8, 6))
ax_hsa = fig_hsa.add_subplot(111, projection='3d')

def update_frame_hsa(frame):
    ax_hsa.clear()
    ax_hsa.scatter(trajectories_hsa[frame][:, 0], trajectories_hsa[frame][:, 1],
\
    trajectories_hsa[frame][:, 2], c='blue', label='HSA')

    ax_hsa.scatter(positions_nanoparticle[:, 0], positions_nanoparticle[:, 1], \
    positions_nanoparticle[:, 2], c='red', label='Nanoparticle')

    ax_hsa.set_title(f'HSA Trajectory - Frame {frame + 1}')
    ax_hsa.set_xlabel('X-axis')
    ax_hsa.set_ylabel('Y-axis')
    ax_hsa.set_zlabel('Z-axis')
    ax_hsa.legend()
    ax_hsa.set_xlim([0, box_size])
    ax_hsa.set_ylim([0, box_size])
    ax_hsa.set_zlim([0, box_size])

ani_hsa = animation.FuncAnimation(fig_hsa, update_frame_hsa, \
frames=num_frames, interval=100)

ani_hsa.save('hsa_trajectory.mp4', writer='ffmpeg')

# Insulin Animation
fig_insulin = plt.figure(figsize=(8, 6))
ax_insulin = fig_insulin.add_subplot(111, projection='3d')

def update_frame_insulin(frame):
    ax_insulin.clear()
    ax_insulin.scatter(trajectories_insulin[frame][:, 0], \
    trajectories_insulin[frame][:, 1], \
    trajectories_insulin[frame][:, 2], c='green', label='Insulin')

    ax_insulin.scatter(positions_nanoparticle[:, 0], positions_nanoparticle[:,
1], \
    positions_nanoparticle[:, 2], c='red', label='Nanoparticle')

    ax_insulin.set_title(f'Insulin Trajectory - Frame {frame + 1}')
    ax_insulin.set_xlabel('X-axis')
    ax_insulin.set_ylabel('Y-axis')
    ax_insulin.set_zlabel('Z-axis')
    ax_insulin.legend()
    ax_insulin.set_xlim([0, box_size])
    ax_insulin.set_ylim([0, box_size])
    ax_insulin.set_zlim([0, box_size])
```

```
ani_insulin = animation.FuncAnimation(fig_insulin, update_frame_insulin, \
frames=num_frames, interval=100)

ani_insulin.save('insulin_trajectory.mp4', writer='ffmpeg')

plt.show()
```
